# Nutritional content and renoprotective potential of miracle tree (*Moringa oleifera*)

**DOI:** 10.5114/bta/204529

**Published:** 2025-06-30

**Authors:** Vibhuti Sharma, Gaytri Mahajan, Reena Gupta

**Affiliations:** Department of Biotechnology, Himachal Pradesh University, Summerhill, Shimla, India

**Keywords:** Moringa oleifera, anti-inflammatory, kidney, superoxide dismutase, catalase, glutathione, pharmacological

## Abstract

Kidney disease is a significant global health issue. This review explores the causes of renal illness, the therapeutic properties of the angiosperm *Moringa oleifera*, and its potential effectiveness in kidney disease management. In chronic kidney disease, inflammatory processes and immune cell activation lead to excessive free radical production, resulting in oxidative stress due to diminished antioxidant capacity. *M. oleifera* possesses diverse health benefits, particularly its ability to enhance antioxidant defenses. Extracts from its stem, seed, and leaf powders have been shown to increase the activity of key antioxidant enzymes, including glutathione, superoxide dismutase, and catalase. Additionally, *M. oleifera* modulates inflammation by reducing the activity of TNF-α, COX-2, and other pro-inflammatory cytokines. This review provides insights into the pharmacological and therapeutic potential of *M. oleifera*, highlighting its promise in the development of novel treatments for kidney-related disorders. Moreover, its bioactive compounds may contribute to renal tissue regeneration and protection against nephrotoxic agents.

## Introduction

Kidney disease is one of the major challenges in global public health. Acute kidney injury (AKI) and chronic kidney disease (CKD) are closely interconnected. Since 1990, CKD has been classified as a noncommunicable disease in the Global Burden of Disease study. The kidney illness has elevated to a global issue as its growth rate quickens. The majority of these events take place in developing and lower-middle-income countries (Romagnani et al. 2017; Webster et al. 2017; Sugahara et al. 2017). In CKD patients, the glomerular filtration rate (GFR) falls below 60 ml/min per 1.73 m^2^, eventually leading to kidney failure (Webster et al. 2017; Romagnani et al. 2017). Individuals at high risk of developing CKD often have a history of ischemic heart disease, diabetes, or hypertension (Vallianou et al. 2019). Currently, there is no definitive cure for renal diseases, and existing treatments focus primarily on symptom management and slowing disease progression.

Herbal medicine has expanded rapidly over the past few decades, gaining popularity in both developing and developed countries due to its natural origin and minimal side effects (Ekor 2014). Apart from allopathy, herbal medicines constitute a significant part of India’s recognized health systems, including Naturopathy, Yoga, Homeopathy, Siddha, Unani, and Ayurveda (Srinivasan and Sugumar 2017). In India, more than 1.1 billion people – over 70% of the population – continue to use nonallopathic medicines (Vaidya and Devasagayam 2007). A substantial proportion of the population in lowincome countries relies on traditional healers and their repertoire of medicinal herbs to meet their healthcare needs. Despite the availability of modern pharmaceuticals, traditional remedies remain widely used due to cultural and historical factors (Ekor 2014). Commercially, these products are also becoming increasingly available, particularly in industrialized nations.

In developed countries, the use of herbal medicines for therapeutic purposes saw a marked increase during the latter half of the 20^th^ century. Herbal remedies are an essential part of “Ayurveda,” the Indian medical system, which remains widely practiced (WHO 2022). The assessment of various plant-based products for their traditional use and medicinal value often leads to the discovery of new and modern therapeutic agents for a wide range of diseases. This knowledge serves as a foundation for developing novel drugs from plant sources. *Moringa oleifera*, a plant from the Moringaceae family, is one such therapeutic herb (Paikra et al. 2017).

*M. oleifera* presents important conceptual and methodological gaps that must be addressed to achieve a more comprehensive understanding of its nutritional and nephroprotective potential. While numerous studies have emphasized its antioxidant, anti-inflammatory, and nephroprotective properties, there is insufficient consensus on the specific bioactive components responsible for these effects or their precise mechanisms of action. Furthermore, although it is classified as a functional food, research on its incorporation into dietary therapies for kidney health remains limited. Many studies employ in vitro and in vivo models with considerable variability in extraction methods, dosages, and study durations, making direct comparisons challenging. Moreover, clinical trials are few and often lack standardized protocols for assessing renal function biomarkers. Addressing these limitations through systematic reviews, meta-analyses, and well-structured clinical trials is essential to establish *M. oleifera* as a safe nutraceutical for kidney health.

Known for its rich nutritional profile and medicinal properties, *M. oleifera* has shown promise in supporting kidney health by reducing oxidative stress, preventing renal stone formation, and enhancing overall renal function. Its phytochemical compounds have demonstrated the capacity to enhance endogenous antioxidant defenses, modulate inflammatory pathways, and strengthen cellular resilience against nephrotoxins. Furthermore, its ability to regulate key kidney function biomarkers – such as serum creatinine, urea, and electrolyte balance – indicates a direct role in maintaining renal physiology. Despite these promising features, comprehensive studies evaluating the efficacy of *M. oleifera* in CKD management remain scarce. This study seeks to address this gap by systematically investigating the nephroprotective potential of *M. oleifera*, focusing on its biochemical and physiological effects on kidney function. By clarifying its mechanisms of action and therapeutic relevance, this research contributes to the expanding body of evidence supporting the integration of *M. oleifera* into renal health management strategies.

*M. oleifera* is one of the most valuable plants, capable of thriving in a variety of environments due to its resilience in harsh conditions such as high temperatures and limited water availability (Trigo et al. 2020). It is indigenous to Northwest India, which remains its primary production region, but it is also found in Madagascar, Northeast Africa, South Africa, Tropical Asia, Latin America, and Southwest Asia (Meireles et al. 2020). Several attributes – including its nutritional value, amino acid profile, and flavone content – have earned *M. oleifera* the moniker “Miracle Tree” and attracted commercial interest. Table 1 presents the taxonomic classification of the *M. oleifera* tree.

**Table 1:** Taxonomical classification of *Moringa oleifera* tree (Kumar and Pareek 2021)

Kingdom	Plantae
Sub kingdom	Tracheobionta
Super division	Supermatophyta
Division	Magnoliophyta
Class	Magnoliopsida
Sub class	Dilleniidae
Order	Capparales
Family	Moringaceae
Genus	*Moringa*
Species	*Oleifera*

*Moringa* leaves and pods release a neurotransmitter called acetylcholine. Additionally, each 100 g serving contains 423 mg of choline. Choline is vital for maintaining normal membrane structure and cellular function. The liver uses it to synthesize various molecules, while the kidneys require it to regulate the body’s water balance. *Moringa* root bark extracts have been shown to enhance renal excretion of calcium and phosphate and to reduce renal stone weight by lowering calcium oxalate and calcium phosphate deposition in the kidneys under conditions of hyperoxaluria induced by propylene glycol. Kidney failure often leads to anemia, which can further worsen renal conditions. The high folic acid and mineral content of *Moringa* enhances its role in facilitating iron absorption. Therefore, regular consumption of *Moringa* may help prevent anemia in renal patients. *Moringa* seeds are also rich in the amino acids methionine and cysteine, which are pro-digestive proteins. These proteins, being essential for digestion, are beneficial for individuals with CKD.

## Morphology of *Moringa oleifera*

The diverse vernacular names of *M. oleifera* across different regions reflect its widespread cultivation and recognition, which are largely influenced by its distinctive morphology — including drumstick-like pods, feathery leaves, and fast-growing nature. Commonly known as the drumstick tree, horseradish tree, or miracle tree, *M. oleifera* has earned various names in different cultural and linguistic contexts. Table 2 lists the vernacular names of *M. oleifera*.

**Table 2:** Vernacular names of *Moringa oleifera* (Koul and Chase 2015)

Language	Vernacular name
Latin	*Moringa oleifera*
English	Drumstick tree, Horseradish tree, Ben tree
Hindi	Saguna, Sainjna
Sanskrit	Subhanjana
Gujarati	Suragavo
Urdu	Sahajna
Tamil	Morigkai
Malayalam	Murinna, Sigru
Telugu	Mulaga, Munaga
Oriya	Munigha, Sajina
Punjabi	Sainjna, Soanjna
Arabian	Rawag
Sindhi	Swanjera
Unani	Sahajan
French	Moringe á graine ailée, Morungue
Spanish	Angela, Ben, *Moringa*
Portuguese	*Moringa*, Moringueiro
Chinese	La ken

**Figure 1:**
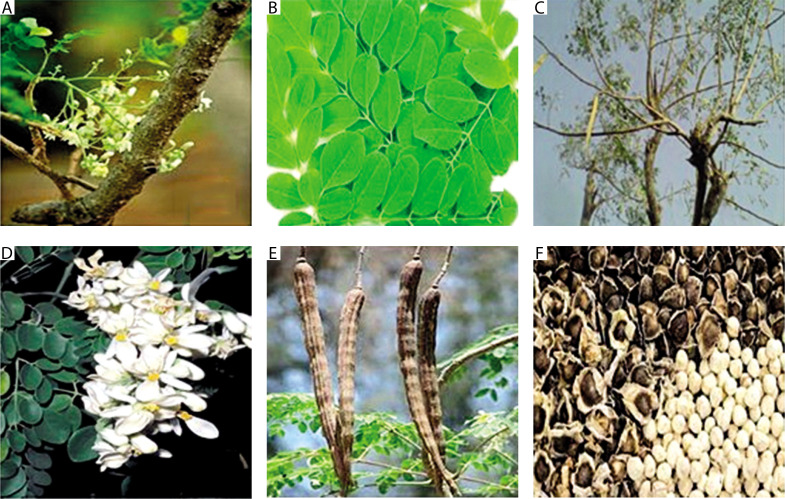
Different parts of the *Moringa oleifera* tree: (**A**) stem, (**B**) leaves, (**C**) branches, (**D**) flowers, (**E**) fruits (pods), and (**F**) seeds

*M. oleifera* is a small to medium-sized tree that can grow up to 10–12 m in height at full maturity. It features a broad, spreading crown that resembles an umbrella. The various parts of the *M. oleifera* tree are depicted in Figure 1.

**Stem:** The stem is generally straight, though it may occasionally appear twisted. It initially grows to a height of 1.5–2 m before branching but can reach up to 3 m.

**Leaves:** The tree bears tripinnate compound leaves that appear airy, with green to dark green elliptical leaflets, each 1–2 cm in length. The alternate, bipinnate, or tripinnate leaves are usually located at the tips of branches. When young, they measure between 20–70 cm in length, are grayish and downy, and consist of 8–10 pairs of pinnae. Each pinna bears two pairs of opposite elliptic or obovate leaflets and one terminal leaflet, each 1–2 cm long (Paliwal et al. 2011).

**Branches:** The canopy is umbrella-shaped, with extended branches that grow in an unorganized manner.

**Flowers:** The flowers are conspicuous, lightly scented, and borne on inflorescences 10–25 cm (4–10 in) long. They are normally white to cream-colored, 2.5 cm in diameter, borne in sprays, with 5 at the top of the flower, and can be tinted with pink in some forms. The flowers which are delightfully scented and 2.5 cm wide, are abundantly produced in auxiliary, hanging panicles 10–25 cm long (Sachan et al. 2010). The flowers also display yellow dots at the base. The five reflexed sepals are linearlanceolate, while the petals are thin and spatulate. All petals, except the lowermost, surround five stamens and five staminodes and are reflexed.

**Fruits/Pods:** The fruits are trilobed capsules commonly referred to as pods. Immature pods are green and may exhibit a crimson tint in certain cultivars. When mature and dry, the pods become pendulous, brown, and triangular, ranging from 30 to 120 cm in length and approximately 1.8 cm in breadth. Each pod typically contains around 20 seeds embedded in the pith. The pod tapers at both ends and is distinctly ribbed (Paliwal et al. 2011).

**Seeds:** The seeds are spherical and encased in a brownish, semipermeable seed shell with three papery wings. Seed hulls are usually brown to black, although they may appear white in low-viability kernels. Viable seeds typically germinate within 2 weeks. The hull features three white wings that run longitudinally from top to bottom at 120° intervals. A single tree may produce 15,000 to 25,000 seeds annually. The average seed weight is 0.3 g, with a kernel-to-hull ratio of 75 : 25 (Mishra et al. 2009).

## Growth conditions for *Moringa oleifera*

*M. oleifera* can be cultivated through stem cuttings, transplanting, or direct sowing (Olson et al. 2019). It is important to note that this crop is relatively easy to grow, as it propagates both sexually and asexually, and requires minimal water and soil nutrients. *M. oleifera* thrives best in semiarid and warm tropical regions due to its drought tolerance, performing well in areas with annual rainfall ranging from 250 to 3000 mm at elevations below 600 m (Masih et al. 2019). However, it has also been successfully cultivated at elevations exceeding 2000 m.

The plant can tolerate poor soils and a wide range of climatic conditions, including droughts, high temperatures, and moderate frosts (James and Zikankuba 2017). The optimal temperature for growth ranges from 25 to 35°C, although the tree can survive temperatures as high as 48°C for short periods (Palada et al. 2019). While *M. oleifera* grows most efficiently in sandy loam and welldrained sandy soils. It can endure clayey soils, but not water buildup for lengthy durations of time because it can stunt development (Nouman et al. 2014). It is an incredibly rapidly growing tree, therefore, due to the high output of the crop, there is significant growth in just 3 months. It typically grows to a height of 5–10 m (Liu et al. 2018).

## Limiting factors for the growth of *Moringa oleifera*

According to various studies conducted by specialists, there are certain limiting factors affecting the growth of this crop:

– *M. oleifera* is highly susceptible to cold temperatures. During the coldest months, it can tolerate temperatures ranging from 1 to 3°C and withstand brief, low-intensity frosts (Trigo et al. 2020). However, prolonged frost exposure can be fatal to the plant. Therefore, low temperatures are considered a major limiting factor in its development.
– For optimal growth and high production of pods and leaves, the plant requires consistently high daily temperatures between 25 and 35°C, making such conditions the most economically viable for cultivation (Godino et al. 2013).
– It cannot survive sustained temperatures above 48°C (Palada et al. 2019).
– The ideal rainfall range, as indicated by isohyets (lines joining points with equal rainfall), falls between 300 and 500 mm annually (Godino et al. 2013).
– The chance of mild frost is low if the average temperature is higher than 8°C, thus the plant may live even though it would not start to develop (Balakumbahan et al. 2020).

Despite these limitations, findings from several experts suggest that *M. oleifera* is a highly adaptable species with rapid growth and strong resilience to adverse climatic conditions. As a result, in the current context of climate change, this crop may serve as a viable alternative to intensive farming systems (Daba 2016).

## Nutritional content in *Moringa oleifera*

Despite having low levels of fat and carbohydrates, *M. oleifera* leaves are an excellent source of protein and essential amino acids, making them a complete nutritional supplement. Furthermore, *M. oleifera* exhibits a rich nutritional profile, providing 205–350 cal/g and containing substantial amounts of protein (19–29%) and dietary fiber (19–37%) (Abou-Zaid and Nadir 2014). Significant concentrations of essential vitamins, minerals, amino acids, and fatty acids are present in both its leaves and seeds. It contains B-complex vitamins, including B_6_, as well as vitamins A, C, and E, along with minerals such as iron, magnesium, and folate. The leaf, pod, and seed extracts also contain vitamins A, B, C, D, E, pyridoxine, folic acid, and nicotinic acid, which act as natural antioxidants (Olagbemide and Alikwe 2014). Plant leaves also include potassium, zinc, magnesium, iron, sodium, calcium, and copper. It is high in proteins, antioxidants, isothiocyanates, and flavonoids (Kou et al. 2018).

**Figure 2:**
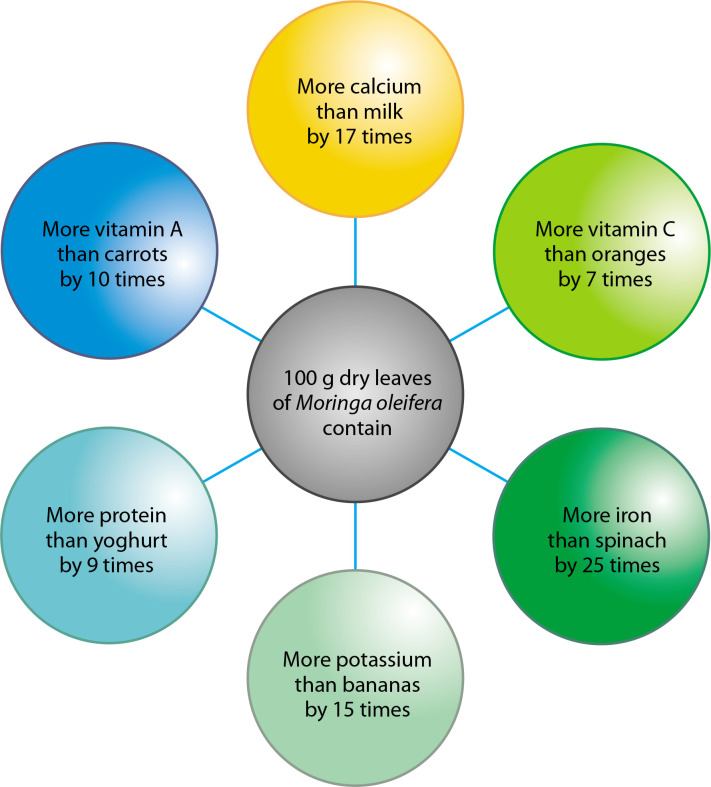
Comparative nutritional content of *Moringa oleifera* with commonly consumed foods such as milk, fruits, and vegetables

The plant is considered nutritionally dense due to the presence of numerous vital compounds in its leaves, seeds, and pods. According to some estimates (Figure 2), *Moringa* contains 25 times more iron than spinach, 10 times more vitamin A than carrots, 17 times more calcium than milk, 9 times more protein than yogurt, and 7 times more vitamin C than oranges. It also contains phytosterols such as sitosterol, campesterol, and stigmasterol – all of which are precursors to hormones (Kou et al. 2018).

### Proteins and minerals

Protein, commonly referred to as a “building block unit,” is a crucial macronutrient for the human body that promotes general growth. The body can synthesize the nonessential amino acids, but it must obtain the necessary amino acids from food sources. The common sources of necessary amino acids include eggs, chicken, fish, and red meat, among others. Even though it provides the most essential amino acids, vegetarians may find it difficult to consume it because most of the proteins derived from plants do not contain an entire profile of essential amino acids.

*Moringa* leaves contain numerous phytochemicals, including tannins, flavonoids, sterols, alkaloids, terpenoids, and saponins (Berkovich et al. 2013). Estimated profiles of *Moringa* fresh leaves, dried leaves, and leaf powder are presented in Table 3.

**Table 3:** Estimate profiles of *Moringa oleifera* fresh leaves, dry leaves, and leaf powder (Islam et al. 2021)

Nutrients	Plant materials, g/100 g		
	Fresh leaves	Dried leaves	Dried leaves powder
Carbohydrates	12	41	38
Fats	2	5	2
Proteins	7	29	27
Fiber	1	12	19

Nearly all parts of the *M. oleifera* plant are utilized in various ways by communities around the world. Fresh *Moringa* leaves can be consumed directly in cooking or preserved in powdered form (Fahey 2005). The seeds are also edible (Berger et al. 1984). Various essential amino acids are found in *Moringa* leaves. Studies have shown that *Moringa* leaves contain amino acids comparable to those found in animal proteins, such as methionine, threonine, lysine, valine, and isoleucine. *Moringa* leaf powder is now used in many excellent products. Table 4 compares the amino acid composition of proteins from conventional sources produced from animals and plants with those from *Moringa* leaf powder.

**Table 4:** Comparison of amino acid profiles of various protein sources and *Moringa* leaf powder (Islam et al. 2021)

Amino acids	Plant derived sources			Animal derived sources	
	Moringa leaf powder	Wheat flour	Soyabean	Egg	Chicken breast
Histidine	2.2	0.23	2.6	2.4	4.5
Isoleucine	4.8	0.311	5.3	6.6	3.24
Leucine	9.2	0.69	7.7	8.8	6.4
Lysine	5.6	0.28	6.4	5.3	7.9
Methionine	1.8	0.22	1.3	3.2	2.5
Phenyl alanine	6.2	0.48	5.0	5.8	3.2
Threonine	4.2	0.30	4.0	5.0	3.7
Tryptophan	-	0.12	1.4	1.7	–
Valine	5.4	0.45	5.3	7.2	3.476
Tyrosine	4.0	0.27	3.7	4.2	3.65
Alanine	5.3	0.56	5.0	–	4.7
Arginine	7.4	0.44	7.4	6.2	5.8
Asparagine	–	–	1.3	11	7.8
Glutamic acid	10.7	3.54	19	12.6	11.2
Glycine	5.3	0.41	4.5	4.2	3.4
Proline	2.9	1.07	5.3	4.2	3.2
Serine	4.1	0.51	5.8	6.9	3.4
Cystine	0.6	0.22	1.9	2.3	1.1

### Antioxidants

The human body typically maintains a balance between oxidants and antioxidants. Reactive oxygen species (ROS) are continuously generated in mammals as a result of environmental stressors encountered in daily life (Hajhashem et al. 2010). In response, the body’s cells produce antioxidants to maintain equilibrium with these free radicals. Any disruption in this balance is referred to as oxidative stress, which may result from various disorders or dysfunctions in normal physiological systems. When cellular damage becomes significant, oxidative stress can contribute to the development of chronic diseases. According to Kattappagari et al. (2015), antioxidants are effective in halting the progression of damage associated with such chronic conditions.

The *Moringa* tree is considered a remarkable source of antioxidants, with a higher production capacity than many typical plant-derived sources. Various phytochemicals are obtained from *Moringa*, including polyphenols and flavonoids, which play a key role in regulating oxidative stress in the kidneys. These compounds function by scavenging free radicals, enhancing antioxidant enzyme activity, and suppressing pro-inflammatory signals. They can activate the body’s natural antioxidant defense systems, including superoxide dismutase (SOD), catalase (CAT), and glutathione peroxidase (GPx), thereby reducing oxidative damage to renal cells. In addition, they inhibit pro-oxidant enzymes such as NADPH oxidase and decrease the activity of nuclear factor-kappa B (NF-κB), ultimately lowering inflammation-induced oxidative stress.

Polyphenols like resveratrol and epigallocatechin gallate have been shown to offer protection against nephrotoxicity caused by toxins, ischemia, and metabolic diseases (Baptista et al. 2024). One study investigated the protective effects of methanolic extract of *M. oleifera* in rats subjected to renal ischemia-reperfusion injury. The results indicated a significant reduction in oxidative stress markers and improvement in renal function, suggesting its potential for mitigating acute kidney injuries (Akinrinde et al. 2020).

In one study, the antioxidant activity of *M. oleifera* was reported to be 21.52%, attributed to its phenolic components (Al-Taweel and Al-Anbari 2019). The antioxidant potential of *Moringa* leaf extract has been demonstrated under both *in vitro* and *in vivo* conditions, primarily due to its rich flavonoid and phenolic content (Khor et al. 2018). Methanolic and ethanolic extracts of *M. oleifera* leaves exhibited the highest antioxidant activity, measured at 65.1% and 66.8%, respectively (Lalas and Tsaknis 2002). The antioxidant properties of *Moringa* leaf powder are known to offer protection against oxidative stress (Wink 2012). Bennett et al. (2003) identified the presence of glycosides, kaempferol, gallic acid, and chlorogenic acid in *Moringa* leaves – compounds known for their antioxidant capabilities. Freeze-dried *Moringa* leaves also retain significant antioxidant activity (Uphadek et al. 2018).

In a recent study, the methanolic extract of *M. oleifera* leaves demonstrated substantial antioxidant activity, which was strongly correlated with its total phenolic content. The study concluded that *M. oleifera* is a potent source of antioxidants due to the presence of ascorbic acid, flavonoids, phenolics, and carotenoids (Peñalver et al. 2022). Given the increasing consumer demand for natural alternatives, there is a significant need for antioxidants derived from natural sources like *Moringa*.

### Vitamins and minerals

In addition to macronutrients such as carbs, proteins, and fats, the human and animal body requires a range of micronutrients to function properly. These micronutrients, which act as carriers or take part in the breakdown of macronutrients, are crucial for the body. Vitamins are crucial since they are essential for the animal body’s ability to process energy. Diseases like beriberi, rickets, scurvy, etc. are relatively frequent and are brought on by vitamin deficiencies. Vitamins include vitamin A (beta-carotene), vitamin B (folic acid, pyridoxine, and nicotinic acid), vitamin C, vitamin D, and vitamin E are all present in *M. oleifera* (Mbikay, 2012). Consequently, processed products made from *Moringa*, such as leaf powder, can serve as valuable dietary sources of essential vitamins.

*Moringa* also contains numerous minerals vital for physiological growth and development. Among these, calcium is one of the most important, and *Moringa* leaf powder is an excellent source – containing up to 17 times more calcium than milk (Gopalakrishnan et al. 2016). In addition, it provides between 25.53 and 31.03 mg/kg of zinc and approximately 2 mg/100 g of iron, which is sufficient to meet daily dietary zinc requirements. A detailed list of vitamins and minerals found in *Moringa* pods, seeds, and leaves is presented in Table 5.

**Table 5:** List of the vitamins and minerals present in pods, seeds, and leaves (Islam et al. 2021)

Nutrients	Plant material (mg/100g)				
	Seeds	Pods	Fresh leaves	Dry leaves	Dry leaves powder
Vitamin B_1_	0.05	0.05	0.06	2.02	2.64
Vitamin B_2_	0.06	0.07	0.05	21.3	20.5
Vitamin B_3_	0.2	0.2	0.8	7.6	8.2
Vitamin C	4.5	120	220	15.8	17.3
Vitamin E	751	–	448	10.8	113
Sulphur	0.05	137	–	–	870
Iron	–	5.3	0.85	25.6	28.2
Copper	5.2	3.1	0.07	0.49	0.57
Potassium	–	259	259	1236	1324
Phosphorus	75	110	70	252	204
Magnesium	635	24	42	448	368
Calcium	45	30	440	2185	2003

## Role of *Moringa oleifera* in kidney disorders

The kidneys play a vital role in maintaining systemic equilibrium within the body. Inflammation is a central pathological feature of kidney disease (Anders and Schaefer 2014; Uddin et al. 2018; Uddin et al. 2021a). Both acute and chronic kidney conditions – such as those caused by toxins, ischemia, or inflammation – affect the renal tubules, leading to renal fibrosis and a progressive decline in GFR (Mackensen-Haen et al. 2021). In response to kidney damage, cytokines are produced, which extend the acute phase of renal disease (Akcay et al. 2009). CKD is also commonly associated with persistent inflammation (Akchurin and Kaskel 2015). Several plant-derived compounds – such as diosmin, hesperidin, fucoidan, thymoquinone, and withaferin – have demonstrated anti-inflammatory effects.

**Figure 3:**
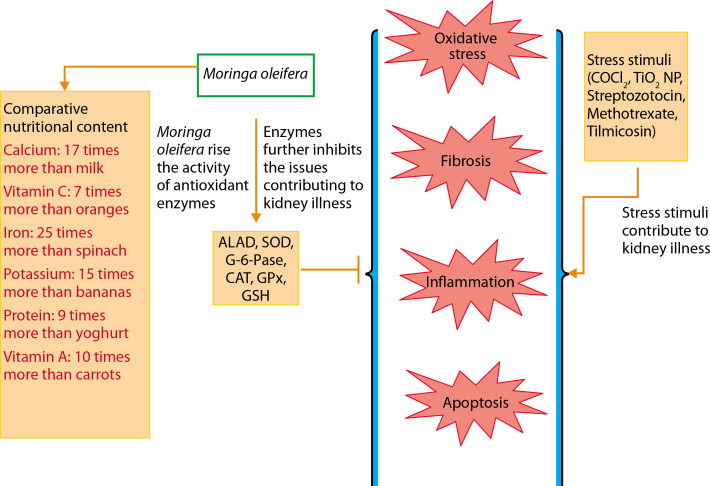
Overall nutritional profile and renoprotective potential of *Moringa oleifera*

The major pathological processes contributing to kidney dysfunction include apoptosis, oxidative stress, fibrosis, and inflammation. The comprehensive nutritional composition and renoprotective potential of *M. oleifera* are illustrated in Figure 3. *M. oleifera* is rich in various bioactive compounds, including isothiocyanates, flavonoids (such as quercetin and kaempferol), and phenolic acids, all of which contribute to its nephroprotective effects. These phytochemicals are known to modulate key cellular pathways involved in renal injury, especially those related to oxidative stress, inflammation, and apoptosis (Akter et al. 2021).

**Nrf-2 pathway activation:** Isothiocyanates, particularly moringin (4-[(α-L-rhamnosyloxy) benzyl] isothiocyanate), inhibit Keap1-Nrf2 binding, thereby activating the Nrf2 pathway. This activation induces the transcription of antioxidant response element (ARE)-regulated genes, including heme oxygenase-1 (HO-1), SOD, catalase, and glutathione peroxidase. These enzymes collectively enhance cellular defense against oxidative stressinduced kidney damage (Abdou et al. 2019).

**NF-**κ**B pathway suppression:** Flavonoids such as quercetin and kaempferol inhibit NF-κB activation, a key mediator of inflammatory responses in renal damage. These compounds prevent IκBα degradation and p65 nuclear translocation, thereby reducing the expression of pro-inflammatory cytokines (TNF-α, IL-6, IL-1β) and adhesion molecules. This suppression ultimately mitigates inflammation-driven renal fibrosis.

**Oxidative stress reduction**: The polyphenols and flavonoids in *M. oleifera* function as direct free radical scavengers, reducing lipid peroxidation and protein carbonylation — both indicators of oxidative damage in renal tissues. These effects are particularly evident through decreased malondialdehyde (MDA) levels and increased glutathione (GSH) levels, which help prevent oxidative injury to kidney cells (Akter et al. 2021).

## Mitochondrial protection and apoptosis inhibition

Quercetin and isothiocyanates regulate mitochondrial apoptosis pathways by modulating Bax/Bcl-2 expression, limiting cytochrome c release, and inhibiting caspase-3 activation. This mechanism helps prevent apoptosis in renal tubular cells.

A summary of the protective effects of *M. oleifera* against kidney diseases is presented in Table 6.

**Table 6:** Summary of safeguarding effects of *Moringa oleifera* against kidney diseases (Akter et al. 2021)

S./No.	Experimental model	Treatment dose	Outcomes	Molecular markers
1.	Gentamicin induced Wister rat	28 days of oral treatment with doses of 100, 200 and 400 mg/kg	Oxidative stress	K^+^ level decrease, plasma creatinine decrease, creatinine clearance increase, MDA decrease and SOD increase
2.	Mice provoked with methotrexate	300 mg/kg body weight taken orally daily for seven days	Apoptosis inflammation and oxidative stress	Reducing urea and creatinine, total protein MDA, increasing SOD and GSH, HO-1, Nrf-2, reducing NF-κB and caspase-9
3.	Toxicity brought on by arsenic in rats	500 mg/kg, given orally, once per day	Oxidative stress	Raise ALAD, raise GSH, lower ROS, increase SOD, raise catalase and lower GSSG
4.	Rat stimulated by glycerol	50 and 100 mg/kg for seven days	Oxidative stress Inflammation	Decrease MPO, decrease creatinine, decrease BUN, decrease NO, decrease H_2_O_2_ decrease AOPPs, decrease MDA, decrease PC, increase PT, increase NPT, reduce KIM-1 and reduce NF-κB
5.	Rat kidney fibroblast cells treated with TGF-β	0, 10, 50 and 100 µg/ml	Fibrosis	Reduce the levels of TRII, Smad4 and phospho-ERK
6.	Rabbit injected with iodide	One dose of 50 mg/kg body weight given orally every day for 27 days straight	Oxidative stress	Reduce MDA, raise GSH, reduce NO, reduce lipid peroxidation and reduce ROS
7.	Beryllium-induced rats	150 mg/kg daily for 5 weeks	Oxidative stress	Increase G-6-Pase activity
8.	Male albino rats induced by TiO_2_ NPs	400 mg/kg orally every day for 60 days	Oxidative stress Inflammation	Reduce TNF-α, NF-κB, KIM-1, and HSP-70 as well as NF-κB and NF-κB-related proteins

TGF-β – transforming growth factor β, TiO_2_ NPs – titanium dioxide nanoparticles, MDA – malondialdehyde, SOD – superoxide dismutase, GSH – glutathione, HO-1 – heme oxygenase-1, Nrf-2 – nuclear factor erythroid 2-related factor 2, NF-κB – nuclear factor-kappa B, ALAD – delta-amino levulinic acid dehydratase, ROS – reactive oxygen species, GSSG – oxidized glutathione, MPO – myeloperoxidase, BUN – blood urea nitrogen, NO – nitric oxide, H_2_O_2_ – hydrogen peroxide, AOPP – advanced oxidation protein products, PC – protein carbonyls, NPT – neopterin, KIM-1 – transmembrane tubular protein, G-6-Pase – glucose-6-phosphatase, TNF-α – tumor necrosis factoralpha, HSP-70 – heat shock protein 70

### Oxidative stress

Renal disease is a significant worldwide health issue. Its late diagnosis, which is only possible in an advanced illness stage, is one of the key problems. The primary cause of the renal disease is the absence of a clinical manifestation in the early stages and the fact that routinely evaluated indicators of renal function are only significantly diminished during the advanced stages of the disease (Tonelli and Dickinson 2020). Even before nitrogenous compounds like creatinine and urea begin to build up in the blood, changes at the molecular level of the renal tissue take place. Many mitochondria may be seen in the renal proximal tubules, which are essential for the energy-intensive reabsorption of water and solutes, and contain numerous mitochondria. These mitochondria are a primary source of ROS, making the kidneys especially susceptible to oxidative stress-related injury. According to Ling and Kou (2018) and Daenen et al. (2019), an imbalance between the production of too many free radicals and the antioxidant defense is what leads to oxidative stress. It has become a diagnostic factor and is frequently seen in CKD (Sohn et al. 2017; Hwang et al. 2019; Uddin et al. 2021b). Acute or chronic kidney damage can create free radicals and prooxidants, which can worsen the condition and contribute to the pathogenesis of issues later on. Prevention may be the best course of action for CKD, although people frequently resist getting screened for it.

**Figure 4:**
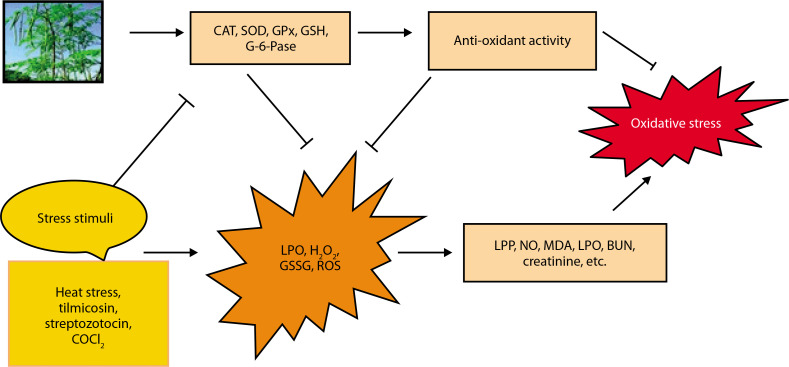
Potential defensive effects against oxidative stress provided by *Moringa*

The potential protective effects of *Moringa oleifera* against oxidative stress are illustrated in Figure 4 (Akter et al. 2021). Exposure to stressors such as streptozotocin, CoCl_4_, methotrexate, tilmicosin, TiO_2_ NPs, and *Salmonella* infection has been shown to elevate levels of oxidative stress markers, including nitric oxide (NO), lipid peroxidation products (LPP), total protein carbonyl content (TPCC), MDA, blood urea nitrogen (BUN), creatinine, and lactoperoxidase (LPO) (Chauhan et al. 2023).

In contrast, *M. oleifera*-treated animals exhibit increased levels of antioxidant markers such as CAT, delta-aminolevulinic acid dehydratase (ALAD), SOD, GPx, GSH, glucose-6-phosphatase (G-6-Pase), and total antioxidant capacity (TAC) (Chauhan et al. 2020). Oxidative stress suppresses the expression of these antioxidant defenses, but GSH has been shown to reduce stress markers such as ROS, H_2_O_2_, oxidized glutathione (GSSG), and LPO. Additionally, GSH supplementation may help mitigate oxidative stress (Akter et al. 2021).

### Fibrosis

A major contributor to kidney fibrosis is the epithelial-to-mesenchymal transition (EMT), primarily regulated by the TGF-β/SMAD signaling pathway and hypoxia (Sohn et al. 2017; Efstratiadis et al. 2009). The antifibrotic effects of *M. oleifera* have been experimentally demonstrated in various models (Park and Chang 2012). *Moringa* root extract significantly reduced TGF-β-induced phosphorylation of ERK and suppressed SMAD4 expression in rat kidney fibroblast cells, suggesting its potential to attenuate renal fibrosis. Additionally, oral administration of *M. oleifera* seed extract was shown to reduce CCl_4_-induced hepatic fibrosis in rats (Hamza 2010). Due to its strong antioxidant, anti-inflammatory, and antifibrotic properties, *M. oleifera* may help prevent fibrosis not only in the kidneys but also in other organs such as the liver. These effects are attributed to its ability to modulate fibrotic signaling pathways and reduce collagen deposition (Hamza 2010).

### Inflammation

**Figure 5:**
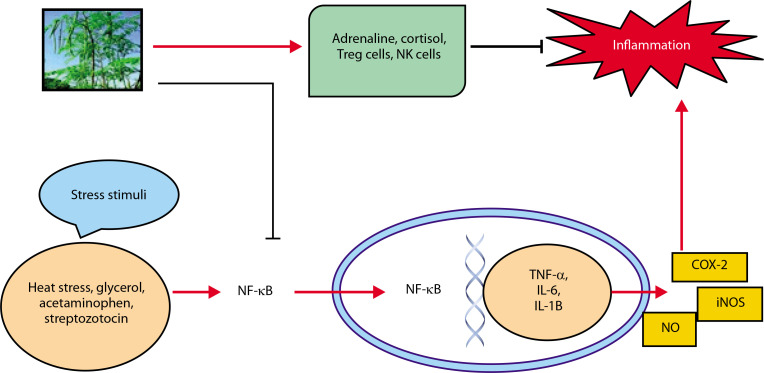
Renoprotective effects of *Moringa oleifera* against inflammation

Inflammation is a key condition that characterizes kidney disease. The kidney is in charge of preserving equilibrium throughout the body (Mackensen-Haen et al. 1981). The acute phase of renal illness is prolonged as a result of kidney damage, which is related to cytokine production levels (Akcay et al. 2009). In addition, chronic inflammation is thought to be a concomitant disease in CKD (Akchurin et al. 2015). Many plants’ active ingredients, including diosmin, withaferin, hesperidin, thymoquinone, fucoidan, etc., exhibit anti-inflammatory properties (Elhelaly et al. 2019; Behl et al. 2020; Abdel-Daim et al. 2020a, 2020b). *M. oleifera* plays a significant role in preventing inflammation-driven kidney damage. Figure 5 shows its renoprotective effects (Akter et al. 2021). In the cytoplasm, production of C-reactive protein (CRP) activates the NF-κB which leads to stress conditions. TNF-α, IL-6, iNOS, IL-1B, and COX-2 are only a few of the proteins that are activated when NF-κB reaches the nucleus and attaches to DNA. These components have all been associated with the development of inflammation. iNOS increases NO production, a known inflammation mediator. *M. oleifera* reduces the expression of NF-κB and CRP in the cytosol and enhances levels of anti-inflammatory agents such as NK and Treg cells, cortisol, and adrenaline.

Adrenaline and the anti-inflammatory chemicals cortisol Treg cells and NK cells are both anti-inflammatory controllers.

TNF-α, IL-6, and MCP-1, an essential chemokine, were down-regulated by the methanol extract of *M. oleifera* to attenuate inflammation in streptozotocin-stimulated male Wister rats (Omodanisi et al. 2017a, 2017b). The ethanol extract of *M. oleifera* reduces the expression of nitric oxide synthase (iNOS) and COX-2, as well as the production of inflammatory indicators, via reducing the phosphorylation of the mitogen-activated protein kinase (MAPK) pathway (Tang et al. 2017). *M. oleifera* leaf extract also inhibits NF-κB activity and the release of pro-inflammatory cytokines (Soliman et al. 2020). In male albino rats exposed to Titanium dioxide nanoparticles (TiO_2_ NPs), the leaf extract reduced levels of KIM-1, thereby preventing interstitial renal inflammation and fibrosis (Abdou et al. 2019).

*M. oleifera* modulates immune responses by enhancing the release of Treg cells, cortisol, adrenaline, NK cells, and leptin (Abdel-Latif et al. 2018). In male Sprague Dawley rats, treatment reduced the expression of TNF-α, TIMP-1, and KIM-1 (Abd-Elhakim et al. 2021). Its seed phytochemicals have been shown to suppress NO production, IL-1 and IL-6 gene expression, and LPSinducible iNOS (Jaja-Chimedza et al. 2017). Flavonoids in *Moringa* inhibit NOS-2 activity and protein tyrosine kinase involved in NOS-2 expression (Olbak et al. 2000; Olszanecki et al. 2002; Sulaiman et al. 2008). Flower extracts of *M. oleifera* can activate toll-like receptors and other pro-inflammatory proteins, but compounds such as quercetin and kaempferol inhibit the NF-κB and STAT-1 pathways (García-Mediavilla et al. 2007; Hämäläinen et al. 2007). Notably, hydroethanolic extract from *Moringa* flowers, particularly at 80% concentration, contains potent anti-inflammatory constituents that act via the NF-B signaling pathway (Tan et al. 2015).

### Apoptosis

**Figure 6:**
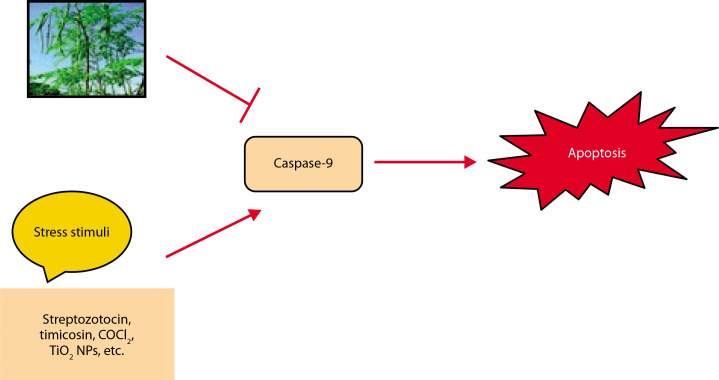
Renoprotective effects of *Moringa oleifera* against apoptosis

Cells are destroyed by a regulated mechanism during apoptosis, a sort of planned cell death. It is a complicated process that depends on energy (Elmore 2007). AKI and even organ failure might result from it (Bonegio and Lieberthal 2002). Ischemia/reperfusion (I/R) causes the kidney to undergo apoptosis or necrosis and lose tubular cells, which lowers GFR (Molitoris 1999; Havasi and Borkan 2011). TNF-cell surface “death receptors,” which cause apoptosis, are expressed in renal tubular cells (Feldenberg et al. 1999). Furthermore, apoptosis is promoted by ROS generation in renal illness (Havasi and Borkan 2011). The renoprotective effects of *M. oleifera* against apoptosis are depicted in Figure 6 (Akter et al. 2021). TNF-α, a well-known inducer of apoptosis, increases the expression of apoptosis-related molecules. However, these effects were significantly suppressed in CoCl_4_-treated rats that received ethanol extracts of *M. oleifera* (Lavey et al. 2011; Abdel-Daim et al. 2020c). In another study, *Moringa* leaf extract administered at a dose of 300 mg/kg body weight reduced the expression of caspase-9, the precursor to caspase-3, thereby decreasing apoptosis (Mace and Riedl, 2010; Soliman et al. 2020). By inhibiting the release of cytochromec and deactivating caspase (Tzifi et al. 2012), Bcl-2 reduced apoptosis while being up-regulated in ML-induced mice by the ethanol extract of *M. oleifera*. TIMP-1, a protein implicated in kidney apoptosis and fibrosis, was also expressed less when *M. oleifera* was present (Abd-Elhakim et al. 2021).

## Other diseases

*M. oleifera* has demonstrated potential as a powerful neuroprotectant. Cerebral ischemia, a condition caused by restricted blood flow to the brain, leads to reperfusion injury and lipid peroxidation, resulting in the generation of ROS. The antioxidants present in *Moringa* can reduce ROS levels, thereby protecting the brain from oxidative damage (Kirisattayakul et al. 2013). *M. oleifera* is also used in the treatment of dementia, as it has been shown to improve spatial memory. Leaf extracts have demonstrated the ability to inhibit acetylcholine esterase activity, thereby enhancing cholinergic function and memory retention (Sutalangka et al. 2013).

Adeyemi and Elebiyo (2014) reported that incorporating *Moringa* into the diet of rats increased protein content and decreased blood urea and creatinine levels, suggesting a protective effect against kidney failure. In studies on gastric ulcers, *Moringa* was shown to reduce stomach acidity by 86.15 and 85.13% at doses of 500 and 350 mg, respectively, indicating its potential use as an antiulcer drug (Choudhary et al. 2013).

Herbal practitioners have also recommended *Moringa* for patients with AIDS. It is advised for inclusion in the diet of HIV-positive individuals to support immune function. However, further research is necessary to determine the interaction of *Moringa* with antiretroviral drugs (Monera and Maponga 2012).

In arthritis studies, a hydroalcoholic extract of *Moringa* flowers significantly reduced the levels of rheumatoid factor, TNF-α, and IL-1 in arthritic rats, suggesting its effectiveness in arthritis treatment (Mahajan and Mehta 2009). Given the prevalence of microbial infections and the rising demand for antimicrobial agents, *M. oleifera* has gained attention for its antibacterial properties (Chen and Verdes 2009). Viera et al. (2010) found that *Moringa* extracts could inhibit the growth of pathogens such as *Bacillus subtilis, Staphylococcus aureus*, and *Vibrio cholera*. These antibacterial effects are attributed to active compounds in the seeds, including pterygospermin, moringine, and benzyl isothiocyanate.

## Conclusion and future perspective

This review highlights the potential of *M. oleifera* in the treatment of renal disorders, including AKI and CKD. Although several studies have demonstrated its nephroprotective effects, the efficacy of its bioactive phytochemicals against renal diseases, particularly through advanced biotechnological techniques. The challenges outlined in this review pave the way for future research to deepen our understanding of natural product-based pharmacological interventions in kidney health.

Furthermore, the development of bioengineered *Moringa* extracts offers promising opportunities for targeted renal therapies. Innovations involving nanotechnology and strategies to enhance bioavailability could significantly improve the therapeutic potential of these extracts. Personalized nutraceutical formulations, guided by metabolomics and nutrigenomics, may enable patient-specific therapies for renal health. Additionally, the formulation of smart functional foods and beverages enriched with bioactive components from *Moringa* could serve as accessible and preventive measures against kidney impairment.

To establish *M. oleifera* as a validated therapeutic option, large-scale randomized controlled trials and epidemiological studies are essential. As a sustainable and renewable resource, bioengineered forms of *M. oleifera* show considerable promise in offering nephroprotective benefits for future renal therapies.
